# Schisandrin B Attenuates Cancer Invasion and Metastasis Via Inhibiting Epithelial-Mesenchymal Transition

**DOI:** 10.1371/journal.pone.0040480

**Published:** 2012-07-25

**Authors:** Zhen Liu, Biao Zhang, Kun Liu, Zonghui Ding, Xun Hu

**Affiliations:** 1 Cancer Institute (Key Laboratory for Cancer Intervention and Prevention of China National Ministry of Education), The Second Affiliated Hospital, Zhejiang University School of Medicine, Hangzhou, China; 2 Second Department of General Surgery, Sir Run Run Shaw Hospital, Zhejiang University School of Medicine, Zhangjiang, People’s Republic of China; Karolinska Institute, Sweden

## Abstract

**Background:**

Metastasis is the major cause of cancer related death and targeting the process of metastasis has been proposed as a strategy to combat cancer. Therefore, to develop candidate drugs that target the process of metastasis is very important. In the preliminary studies, we found that schisandrin B (Sch B), a naturally-occurring dibenzocyclooctadiene lignan with very low toxicity, could suppress cancer metastasis.

**Methodology:**

BALB/c mice were inoculated subcutaneously or injected via tail vein with murine breast cancer 4T1 cells. Mice were divided into Sch B-treated and control groups. The primary tumor growth, local invasion, lung and bone metastasis, and survival time were monitored. Tumor biopsies were examined immuno- and histo-pathologically. The inhibitory activity of Sch B on TGF-β induced epithelial-mesenchymal transition (EMT) of 4T1 and primary human breast cancer cells was assayed.

**Principal Findings:**

Sch B significantly suppressed the spontaneous lung and bone metastasis of 4T1 cells inoculated s.c. without significant effect on primary tumor growth and significantly extended the survival time of these mice. Sch B did not inhibit lung metastasis of 4T1 cells that were injected via tail vein. Delayed start of treatment with Sch B in mice with pre-existing tumors did not reduce lung metastasis. These results suggested that Sch B acted at the step of local invasion. Histopathological evidences demonstrated that the primary tumors in Sch B group were significantly less locally invasive than control tumors. In vitro assays demonstrated that Sch B could inhibit TGF-β induced EMT of 4T1 cells and of primary human breast cancer cells.

**Conclusions:**

Sch B significantly suppresses the lung and bone metastasis of 4T1 cells via inhibiting EMT, suggesting its potential application in targeting the process of cancer metastasis.

## Introduction

Breast cancer is the leading threat to women, with an estimated 1.4 million new cases and 458,000 deaths in 2008 worldwide [1]. For the last two decades, integrations of surgery, chemotherapy, radiation, menopausal hormone therapy, and anti-HER2 drugs contribute considerably to the mortality decline [2]. Nevertheless, despite the significant progresses, distal metastasis of breast cancer to visceral organs and bones is still poorly resolved [1], [3], [4].

Cancer metastasis is a multi-step process that includes local invasion, intravasation to the lymph and blood systems, survival in the bloodstream, extravasation from the microvessels and colonization at a secondary site [5], [6]. Numerous studies have revealed the insight into the mechanisms of each step of cancer metastasis [7], [8]. The multistep of cancer metastasis and the understanding of the associated mechanisms provide numerous opportunities for chemical interventions [9].

**Figure 1:**
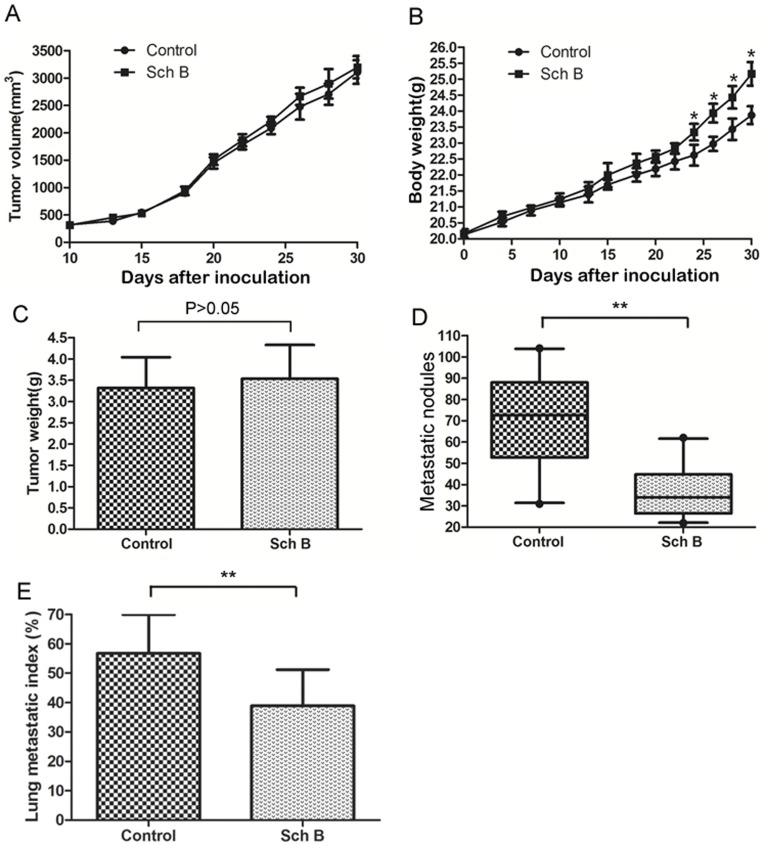
Sch B reduces 4T1 lung metastasis but does not affect the growth of primary tumor. 5×10^4^ viable 4T1 cells were inoculated *s.c.* in the right second mammary fat pad area to establish the spontaneously metastatic model. Sch B (100 mg/kg body weight) or vehicle (0.5% paraxamer) was dosed intragastrically every day for a total of 7 doses. Mice (n = 20 for each group) were sacrificed on day 30. (A) The growth curves of primary tumor (B) Mouse body weight. (C) Primary tumor weight on the day sacrificed. (D) Numbers of visible lung metastatic nodules. (E) The lung metastatic index. *, P<0.05, **, P<0.01.

Schisandrin B (Sch B) is the most abundant dibenzocyclooctadiene lignan present in the traditional Chinese medicinal herb *Schisandra chinensis* (*Turcz.*) *Baill*. In the past several years, Sch B has been revealed to possess multiple functions against cancer. We and others previously reported that Sch B was a dual inhibitor of P-glycoprotein and multidrug resistance protein 1 (MRP1) [10], [11], [12], [13], [14], [15]. Sch B could also enhance doxorubicin-induced apoptosis in cancer cells, through activation of mitochondrial apoptotic pathway, without obvious enhanced toxicities toward normal cells [16], mitigate doxorubicin-induced acute and chronic cardiotoxicity [17], [18], attenuate cisplatin-induced oxidative stress, genotoxicity and neurotoxicity [19], and inhibit ATR protein kinase activity in response to DNA damage [20]. In a pilot study that was intended to observe the effect of Sch B on enhancing the anti-tumor effect of doxorubicin on murine breast cancer 4T1 cells, we noticed that Sch B alone could significantly reduce the spontaneous lung metastasis of 4T1. This preliminary observation prompted us to carry out this study to investigate the inhibitory effect of Sch B on 4T1 in vivo metastasis.

**Figure 2:**
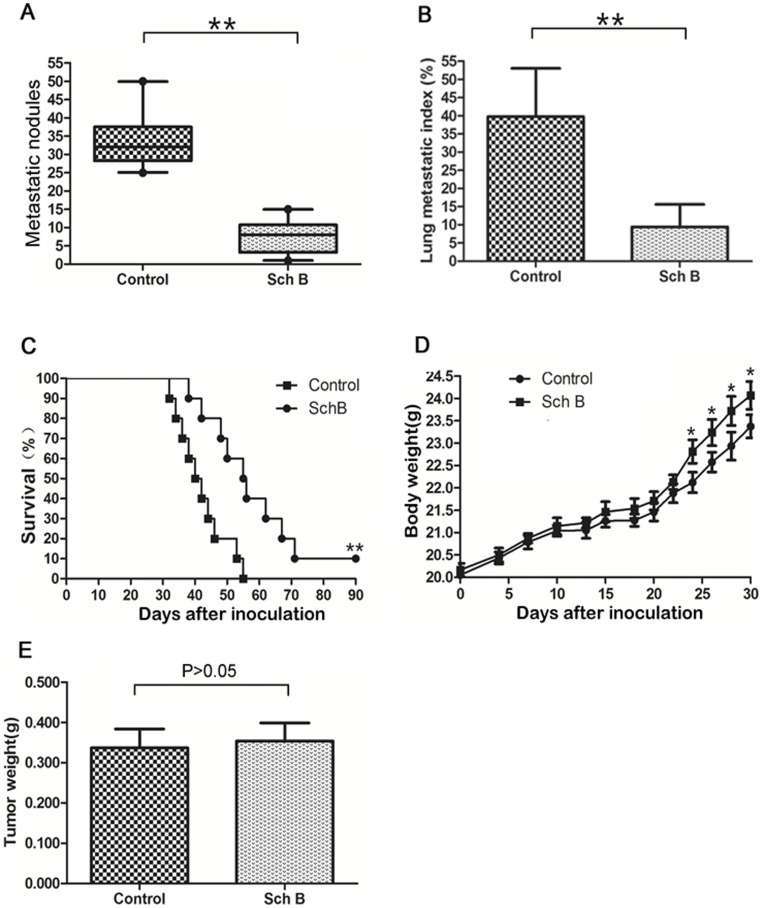
Sch B reduces lung metastasis and prolongs mice survival time. 5×10^4^ viable 4T1 cells were inoculated *s.c.* in the second right mammary fat pad area to establish the spontaneously metastatic model. Mice were treated with Sch B (100 mg/kg body weight) or vehicle every day for a total of 7 doses. Primary tumors were resected on day 10. Two thirds of the mice (n = 20 for each group) were sacrificed on day 30 and one third of the mice (n = 10 for each group) were for the survival experiment. (A) Numbers of visible 4T1 lung metastasis. (B) Lung metastatic index calculated by H&E staining. (C) Mouse survival time. (D) Mouse body weight. (E) Tumor weight resected on day 10. *, P<0.05, **, P<0.01.

**Figure 3:**
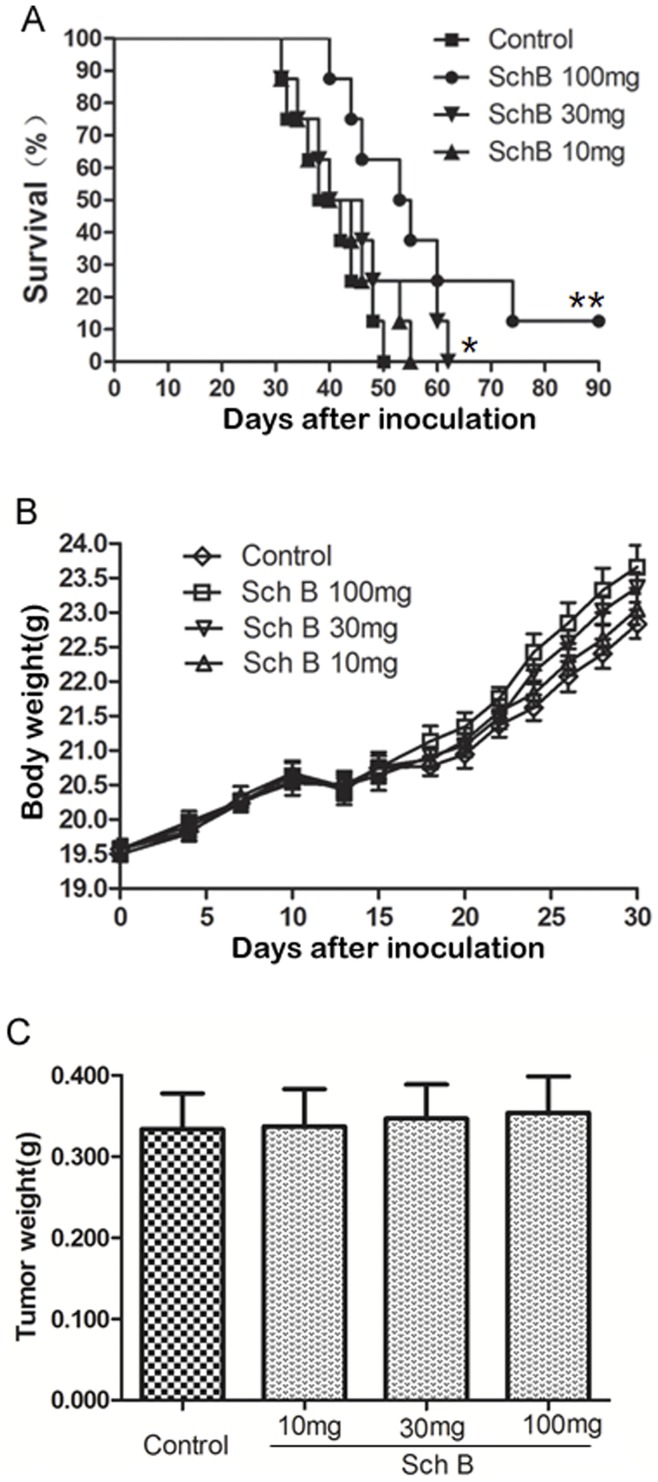
Sch B prolongs mouse survival time in dose-dependent manner. Mice (n = 8 for each group) receiving Sch B (100, 30, 10 mg/kg body weight) or vehicle every day for total 7 doses, and primary tumors were resected on day 10. (A) Mouse survival time. (B) Mouse body weight. (C) Tumor weight resected on day 10. *, P<0.05, **, P<0.01, Sch B versus control group.

## Materials and Methods

### Chemicals and Antibodies

Sch B was from the National Institute for the Control of Pharmaceutical and Biological Products (Beijing, China). Recombinant human tumor growth factor-β1 (TGF-β) was purchased from PeproTech Inc. (Rocky Hill, NJ). Epithelial-mesenchymal transition antibody sampler kit (#9782, including E-cadherin, Snail, Slug & ZEB1 antibodies) was procured from Cell Signaling Technology (Beverly, MA). Vimentin (#550513) and fibronectin (#610078) antibodies were products of BD Biosciences (Franklin Lakes, NJ). Rhodamine phalloidin (#R415) was bought from Invitrogen Corporation (Carlsbad, CA).

**Figure 4:**
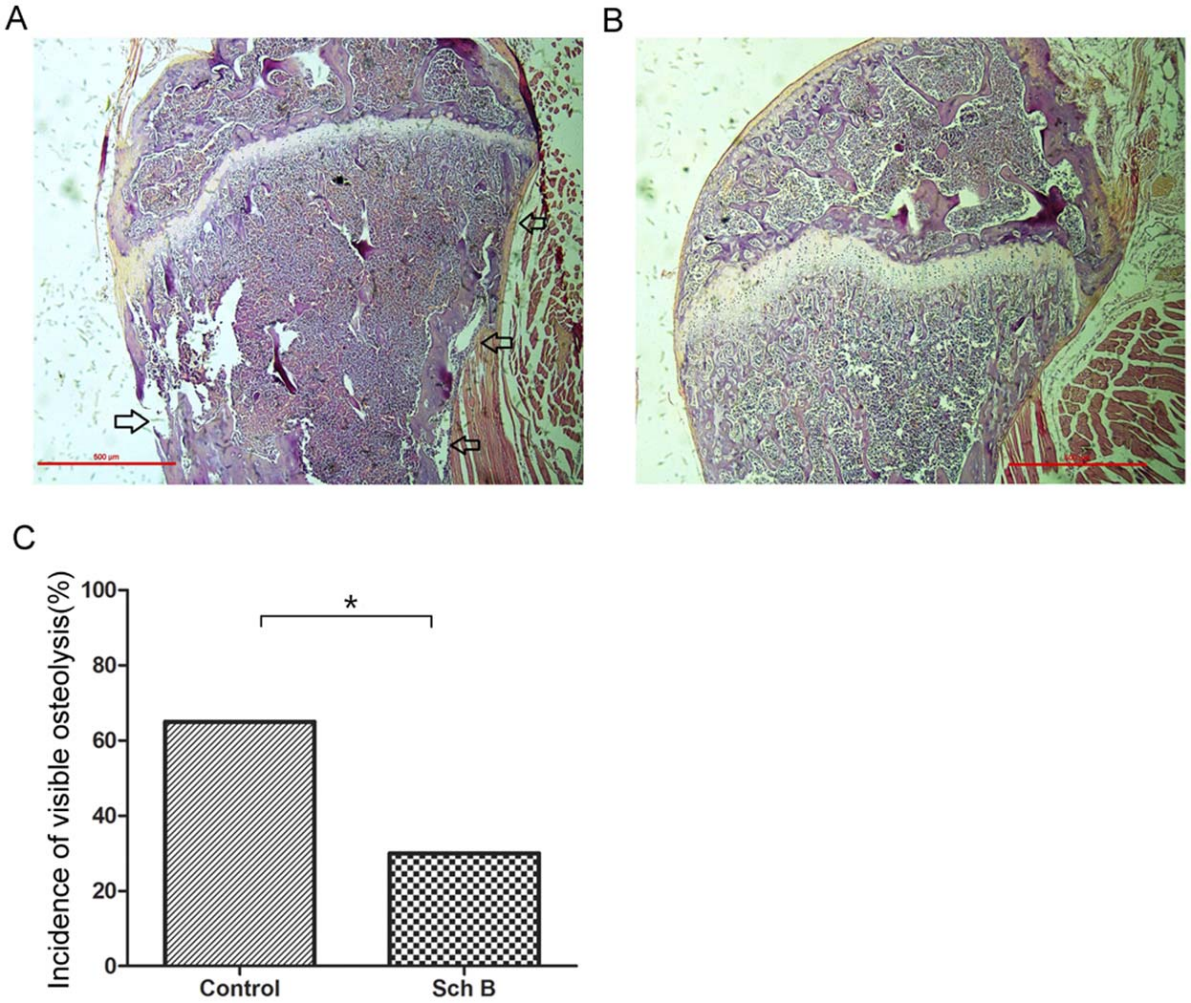
Sch B inhibits bone metastasis of 4T1 cells. Bone metastasis of 4T1 in mice demonstrated in Figure 2 were evaluated using H&E staining as described in Materials and Methods. The incidence of bone metastasis was reflected by the visible osteolysis. (A) Representative photo of bone metastasis. Osteolysis was marked by arrow. (B) Representative photo of bones with no osteolysis. (C) Incidence of bone metastasis (n = 20) compared using chi-square analysis. *, P<0.05, Sch B versus control group.

### Cell Culture

4T1 cells were from the Chinese Academy of Sciences Cell Bank of Type Culture Collection (Shanghai, China) and cultured in monolayers in RPMI-1640 supplemented with 10% fetal bovine serum (FBS), 100 µg/ml penicillin and 100 µg/ml streptomycin. MDA-MB-231 cells were purchased from American Tissue Culture Collection (No. HTB-26; ATCC, Manassas, VA) and maintained in Leibovitz’s L-15 medium with 10% FBS, 100 µg/ml penicillin and 100 µg/ml streptomycin (all from Invitrogen). Both cells were maintained in a humidified incubator at 37°C 5% CO_2_ atmosphere.

**Figure 5:**
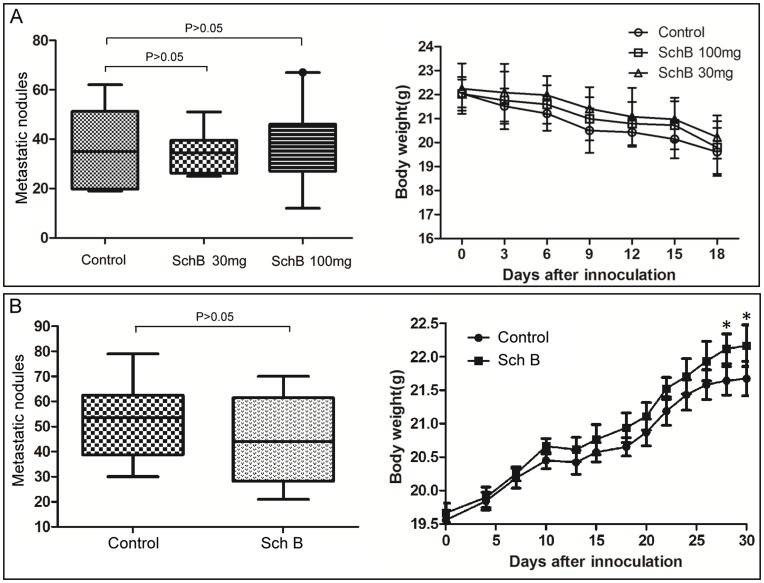
Sch B does not inhibit lung metastasis of 4T1 cells using a tail vein injection or a delayed treatment model. (A) Tail vein injection model: mice (n = 10 for each group) were gavaged with Sch B (daily doses: 30 or 100 mg/kg body weight or vehicle) for 3 days prior to 4T1 cell inoculation. Then, each mouse was inoculated with 2×10^5^ 4T1 cells via tail vein injection, followed by treatment with Sch B (daily doses: gavaged with 30 or 100 mg/kg body weight or vehicle) for the next consecutive 7 days and sacrificed on day 18. (B) Delayed treatment model. Mice (n = 10 for each group) were inoculated with 5×10^4^ 4T1 cells *s.c*. into the second right mammary fat pad area, and the primary tumors were resected on day 10. Sch B (100 mg/kg body weight) or vehicle was given every day from day 13–19 for a total of 7 doses, and mice were sacrificed on day 30. The lung metastasis was scored by macroscopic metastatic nodule as described in Materials and Methods. *, P<0.05, Sch B versus control group.

### Primary Cell Culture

Fresh breast tumors were obtained from patients undergoing surgery with written informed consent. Specimens of infiltrating ductal breast carcinoma without chemotherapy were selected for cell culture as describe by Speirs et al. [21]. The cells were cultured in HuMEC Ready Medium (Invitrogen) supplemented with 2% FBS in a humidified incubator at 37°C 5% CO_2_ atmosphere.

**Figure 6:**
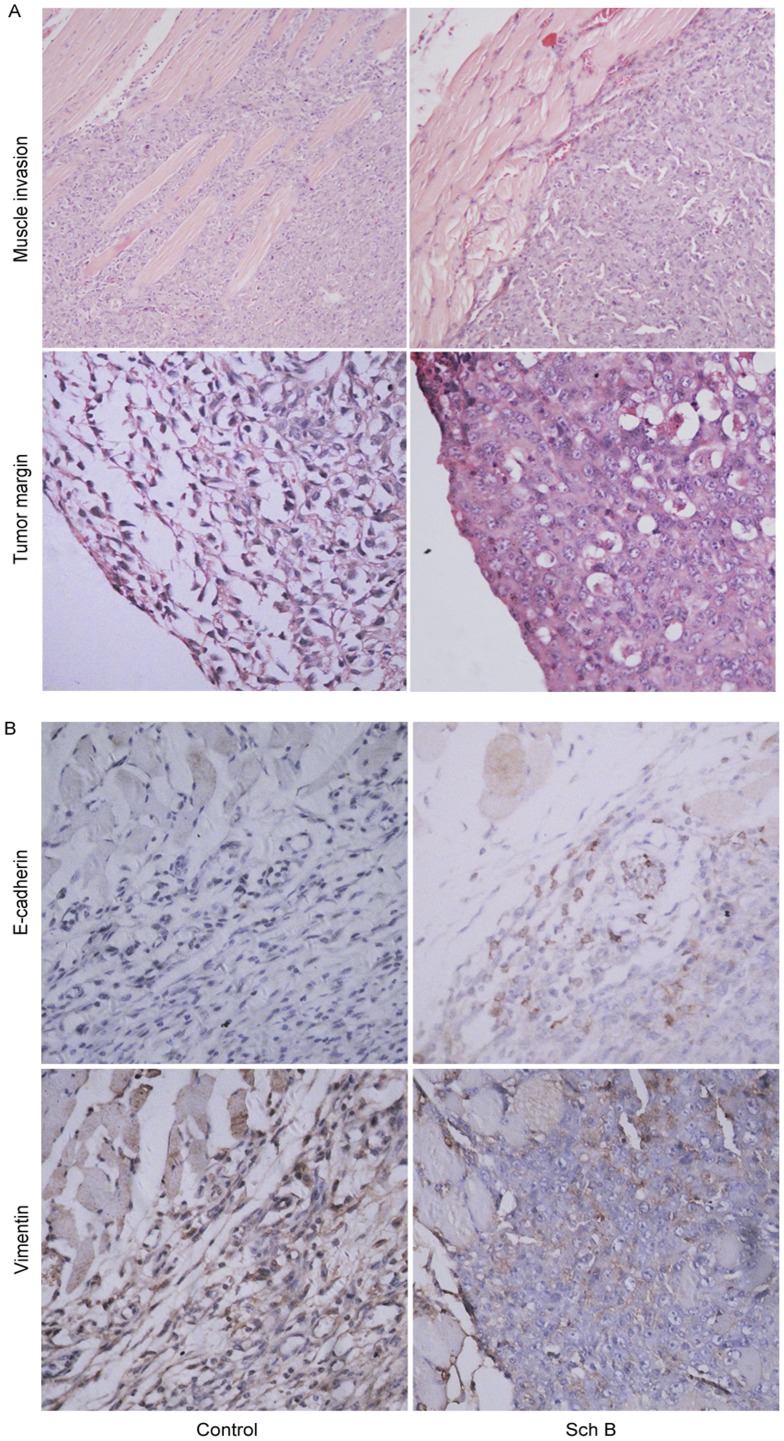
Sch B inhibits local invasion of 4T1 cells *in vivo*. Mice (n = 20 for each group) were treated with Sch B (100 mg/kg body weight) or vehicle intragastrically daily for 7 days. (A) Primary tumors and surrounding tissues were resected on day 15 for hematoxylin-eosin stain (upper, ×40, lower, ×100). (B) immunostaining of E-cadherin and vimentin.(×100).

### Animals

Female BALB/c and BALB/c nu/nu mice (18–22 g) were obtained from Shanghai SLAC Laboratory Animal Co., Ltd (Shanghai, China; Animal certificate: No. SCXK [Hu] 2007–0005). Animals were housed in a standard polypropylene cage containing sterile bedding under a controlled condition of temperature (23±2°C), humidity (50±5%), and light (10 and 14 h of light and dark) in Zhejiang University Laboratory Animal Center (No. SYXK [Zhe] 2007–0029).

### 4T1 Syngeneic Xenograft Model

The animal model is based on the previous report [22]. Each mouse was inoculated with 5×10^4^ viable 4T1 cells *s.c*. in the second right mammary fat pad area. Mice were randomly assigned to Sch B or control group the next day after inoculation. Each mouse was gavaged with either Sch B (100, 30, or 10 mg/kg body weight) or vehicle (0.5% paraxamer, Sigma) every day for a total of 7 doses. To investigate the effect of Sch B on 4T1 metastasis, we used 3 protocols. First, mice were gavaged with Sch B (100 mg/kg body weight), and tumors grew uninterruptedly. Second, mice were dosed with Sch B (100 mg/kg body weight) and then primary tumors were resected on day 10 as previously described [23]. Third, mice were administered with three doses of Sch B (100, 30, or 10 mg/kg body weight) and primary tumors were excised on day 10. The tumor volume was measured by vernier calipers twice or thrice weekly and calculated by an ellipse volume formula V =  (L×W^2^)×0.5, where L is length and W is width. Lung metastases were scored by counting the macroscopic metastatic nodules based on the previous reports [24], [25], and the average number of lung metastases per mouse was determined as an indicator of general cancer disseminations [23], [26].

**Figure 7:**
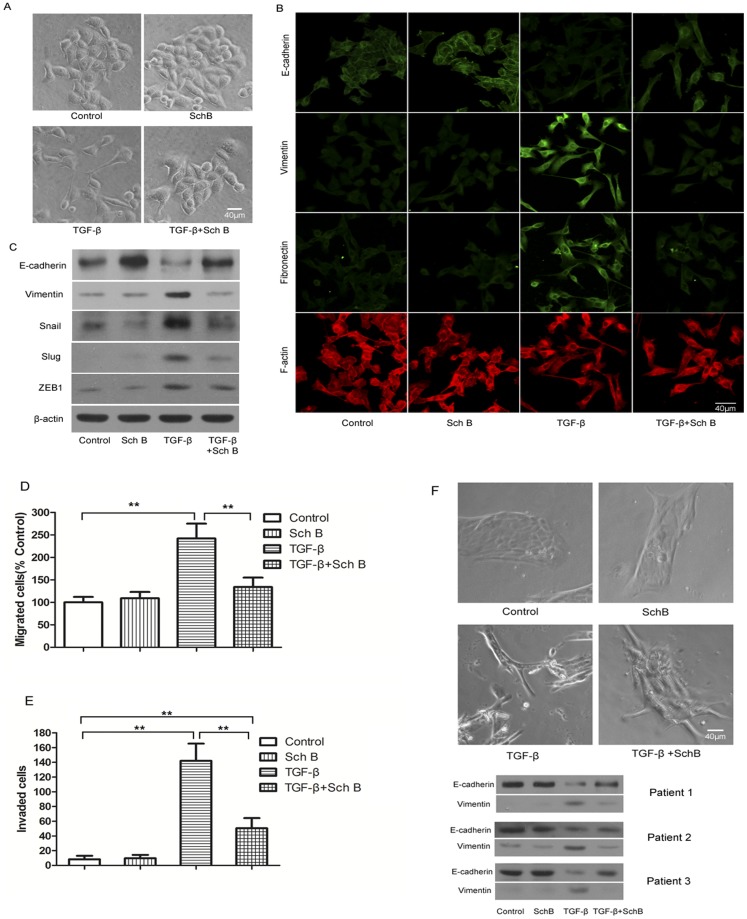
Sch B inhibits TGF-β induced EMT of 4T1 and primary human breast cancer cells *in vitro*. Cells were treated with Sch B (5 µg/ml) and TGF-β (5 ng/ml) as described in Materials and Methods. (A) Representative photos of TGF-β induced morphological change of 4T1 cells with or without pretreatment of Sch B. (B) Fluorescence immunostaining of cellular E-cadherin, vimentin, fibronectin, and F-actin. (C) Western blotting of E-cadherin, vimentin, Snail, Slug, ZEB1, with β-actin as the internal control. (D) and (E), TGF-β induced migration and invasion of 4T1 cells with or without pretreatment of Sch B. (F) Representative photos of TGF-induced the morphological change of primary breast cancer cells with or without pretreatment of Sch B and western blotting of E-cadherin and vimentin of primary breast cancer cells from three individual patients.

### Delayed Treatment Model

Primary tumors were resected on day 10, and Sch B (100 mg/kg body weight) or vehicle was gavaged on day 13–19. All animals were sacrificed on day 30 for data collection.

### Tail Vein Injection Model

This in vivo model is based on the previous report [27]. Before inoculation with 4T1 cells, each mouse was gavaged with Sch B (30, 100 mg/kg) or vehicle (0.5% paraxamer) for 3 consecutive days. Each mouse was then inoculated with 2×10^5^ cells intravenously via tail vein injection, followed by Sch B or vehicle treatment for the next 7 consecutive days. All mice were sacrificed on day 18.

### MDA-MB-231 in vivo Animal Model

Each mouse was inoculated with 5×10^6^ MDA-MB-231 cells *s.c*. in the second right mammary fat pad area. Mice were randomly assigned to Sch B or control group the next day after inoculation. Each mouse was gavaged with either Sch B (100 mg/kg body weight) or vehicle (0.5% paraxamer, Sigma) for the next 7 consecutive days with a total of 7 doses. Mice were sacrificed on day 30 after inoculation.

### Criterion to Assess the Mouse Moribund Status

A moribund mouse is defined as one which exhibits more than one of the following six clinical signs: inability to eat or drink; severe lethargy (reluctance to move when gently prodded with forceps); severe balance or gait disturbance, according to the NIH criterion [28].

### Histological Analyses

Tumor tissue was fixed, embedded, and sliced into 5 µm thick sections. Paraffin sections were stained with hematoxylin and eosin (H&E) (Sigma). For immunohistochemistry, immunostaining of E-cadherin and vimentin was carried out using a standard protocol [29]. The H&E sections were evaluated and photographed with a Nikon Eclipse 50i microscope controlled by the NIS-Elements software (version 3.22). The relative area of metastases was measured using the ImageJ 1.45S software (NIH) [30]. Lung metastasis was quantified by counting the total tissue area per lung section (D1) and metastasis present in the same area (D2). The metastatic index was calculated by the ratio D2/D1 [31]. For bone metastasis, leg bones (femora and tibiae) were fixed in 10% neutral buffered formalin, decalcified in 1% HCl for 24 hours, and embedded in paraffin. Sections were stained with H&E and were examined microscopically. The incidence of bone metastasis was reflected by the visible osteolysis [32].

### Cell Toxicity of Sch B on 4T1 Cells

2×10^3^ cells were seeded per well in 96-well plates. Sch B (5, 10, 20, 40, 80, 160 µg/ml) was added and cultured for 60 hours, cell viability was estimated by trypan blue exclusion. Six wells were measured for each concentration. Half inhibitory concentration (IC_50_) was determined.

### Effect of Sch B on TGF-β Induced EMT

The murine 4T1 cells and human primary breast cancer cells were pretreated with Sch B (5 µg/ml) for 24 hours, then TGF-β (5 ng/ml) was added and cultured for another 36 hours. The cell lysates were used in the western blotting analysis as previously described [33]. Fluorescence immunostaining was performed according to our previous reported method [34].

### Cell Migration and Invasion Assays

For migration assays, 2×10^5^ cells were plated in six-well plates. After pretreatment of Sch B (5 µg/ml) for 24 hours, the FBS concentration of the culture medium was changed to 2% and a wound was incised as previously described [35]. Then TGF-β (5 ng/ml) was added and cultured for another 24 hours, photographs were taken by phase-contrast microscopy. Transwell (Corning Costar, Cambridge, MA) with a filter coated with Matrigel (BD Biosciences) was used for invasion assays. 4×10^3^ cells in serum-free RPMI-1640 were seeded into the 24-well transwell upper chamber, the lower chambers were filled with RPMI-1640 supplemented with 10% FBS as a chemo-attractant. After 12 hours pretreatment of Sch B (5 µg/ml) in both upper chamber and lower chamber, TGF-β (5 ng/ml) was added and treated for another 12 hours. At the end of the assays, the filter side of the upper chamber was cleansed with a cotton swab and the filter was stained for one hour with crystal violet (Sigma) in 2% ethanol and then rinsed in water. Then, cells on the filter were counted in at least 10 fields under the view of a phase-contrast microscope.

### Statistical Analysis

Data were expressed as the Mean ± SD. Mouse survival was evaluated by Kaplan–Meier analysis and the log rank test. Chi-square analysis was used for bone metastasis comparison. Student t tests were used for the comparison between two mean values. P values less than 0.05 were considered statistically significant.

### Ethics Statement

The animal study was approved by the institutional animal ethical committee of Zhejiang University with approval No. zju-2009-1-01-028. Human tumors were obtained with written informed consent by the patients and following a protocol approved by the Ethics Committee of the Second Affiliated Hospital of Zhejiang University.

## Results

### Sch B Significantly Reduces 4T1 Lung Metastasis without Inhibition on Primary Tumor

4T1 syngeneic xenograft model is a widely used to study the spontaneous metastasis of cancer [36]. The major metastatic site of this model is lung [22]. Using this model, we found that Sch B significantly reduced 4T1 lung metastasis but without significant inhibition on primary tumors (Fig. 1).

### Sch B Significantly Increases Survival Time of Mice Via Inhibiting 4T1 Lung Metastasis

To exclude the effect of the primary tumor on mice survival time, we removed primary tumor by surgical resection on day 10 after 4T1 inoculation. Then two experiments with different purposes were performed on these mice. Experiment 1 was to observe the lung metastasis. The mice were sacrificed on day 30 after inoculation and lung metastasis was examined. Results showed that Sch B significantly inhibited 4T1 lung metastasis (Fig. 2 A & B & Fig. S1). Experiment 2 was to observe survival time. Fig. 2C demonstrated that mice receiving Sch B lived significantly longer than control and this effect was dose dependent (Fig. 3A). The growth curves of mice and weight of tumors were comparable between Sch B and control group (Fig. 2D&E, Fig. 3B&C).

It is noted that 10% of mice receiving Sch B (100 mg/kg body weight, Fig. 2C&3A) survived for 3 months, after which the experiment was terminated. This phenomenon was observed in three independent experiments. These mice were executed and examined pathologically. There were no visible lung metastasis in these mice (data not shown). It has been demonstrated by Pulaski and Demaria [37], [38] that metastasis of 4T1 syngeneic tumor could occur at very early stage (e.g., within the first week after s.c. inoculation), and our study suggests that Sch B could interfere with early metastasis. Consistently, we showed that Sch B could attenuate 4T1 local invasion in vivo and inhibit EMT in vitro (see below). The results suggest that early surgery (e.g., surgery done within 10 days after inoculation) combined with Sch B treatment may completely block the in vivo metastasis of 4T1 cells.

### Sch B Attenuates 4T1 Metastasis to Bone

The 4T1 mouse model is commonly used as a model of mammary cancer metastasis to bone [32], [39]. Inhibiting mammary tumor metastasis to the lung may divert tumor cells to metastasize to alternative organs such as bone [32]. In order to rule out this possibility, we investigated if Sch B would divert 4T1 to bone metastasis. Our data demonstrated that Sch B significantly reduced 4T1 metastasis to bone (Figure 4), as reflected by the severity of osteolysis.

### Sch B Inhibits Local Invasion of 4T1 Cells

Although the above results indicated that Sch B could inhibit 4T1 lung and bone metastasis, it was not known how Sch B interfered with 4T1 metastasis. Since cancer metastasis is a complex process involving local invasion, intravasation, survival in the bloodstream, extravasation and colonization at a secondary site, it would be important to identify at which step Sch B interferes with 4T1 metastasis. We thought that the model via tail vein injection of cancer cells may help to tell if Sch B disturbs metastasis at step of the local invasion or not. If Sch B does not inhibit lung metastasis of 4T1 cells injected via tail vein, it should interfere with 4T1 metastasis at the step of local invasion. The results showed that Sch B had no significant inhibitory effects on lung metastasis of 4T1 cells via tail vein injection (Fig. 5A).

In order to further confirm if Sch B affect 4T1 metastasis at the step of early stage, we performed an experiment of delayed treatment with Sch B in mice with pre-existing tumors. The results showed that delayed treatment with Sch B had no significant effect on 4T1 metastasis (Fig. 5B).

Based on the above results, we performed the histopathological examination. As shown in Fig.6A, tumor cells had invaded deeply into the muscles in most of the primary tumors in control group (13 out of 20), while in Sch B group, most tumors showed a clear borderline between the region of tumor cells and muscles (14 out of 20). In addition, cells in the tumor margin in control group were spindle-shaped, more like mesenchymal cells, whereas in Sch B group, tumor cells kept “cobblestone” morphology, more like epithelial cells. These observations suggested that Sch B might inhibit epithelial mesynchymal transition (EMT).

Besides morphological changes, EMT is also accompanied with expressional changes of some biomarkers, such as E-cadherin, vimentin, etc [6], [40], [41]. When cells are under this transition, E-cadherin would be downregulated and vimentin upregulated. We examined the expressions of E-cadherin and vimentin in tumors. Fig.6B showed tumor cells in Sch B group had a higher E-cadherin expression (an epithelial biomarker) but lower vimentin expression (a mesenchymal biomarker) than those in control, suggesting that Sch B inhibited 4T1 metastasis likely via targeting EMT.

Apart from 4T1 model, we also investigated the effect of Sch B on in vivo metastasis of human breast cancer cell line MDA-MB-231 (Fig. S2). 30 days after inoculation, mice were sacrificed. We found that there were neither macroscopic metastatic nodules in lung nor bone metastasis in control and Sch B treated mice. The reports on metastatic ability of this cell line from different laboratories seems mixed: while several groups reported a poor metastatic ability of this cell [42], [43], [44], others [45],via vigorous selection, acquired several subclones (bone-seeking MDA-231BO and brain-seeking MDA-231BR) with high metastatic ability. Nevertheless, despite no obvious metastasis, we noticed that, similar to 4T1 cells, MDA-MB-231 cells invaded deeper into the muscles in most of the primary tumors in control than in Sch B treated group.

### Sch B Inhibited TGF-β Induced EMT of 4T1 and Primary Human Breast Cancer Cells *in vitro*

TGF-β is a strong EMT inducer that can aggravate tumor cell migration & invasion *in vitro* [46]. In the EMT inhibition assay, 5 µg/ml Sch B, a concentration far below its IC_50_ toward 4T1 cells (Fig. S3), was used.

4T1 cells became spindle-shaped or mesenchymal-like after TGF-β treatment, accompanied by a decreased E-cadherin expression and increased expressions of vimentin/Slug/Snail/ZEB1. This effect was significantly attenuated by pretreating cells with Sch B (Fig. 7A, 7B, 7C).

Consistently, in wound-healing assay, TGF-β increased the migration rate of 4T1 cells by about two folds more than that of control, which was as well inhibited by pretreating cells with Sch B (Fig. 7D, Fig. S4A). Similarly, the invasion ability of 4T1 cells enhanced by TGF-β was significantly attenuated by Sch B (Fig. 7E, Fig. S4B), which alone affected neither cell migration nor invasion.

Sch B also inhibited the effect of TGF-β on the primary human breast cancer cells (Fig. 7F).

## Discussion

As cancer related mortality is largely caused by cancer metastasis, to reduce metastasis is a major strategy to combat cancer. It is proposed by a group of well-known scientists that ‘given the fact that primary tumors can often be controlled using conventional therapies, could agents that act specifically on the process of metastasis be more likely to increase long term patient survival’ [47]. In this study, we demonstrate (1) Sch B significantly attenuates 4T1 metastasis thereby extending the survival time of mice; (2) Sch B attenuates 4T1 metastasis at the step of local invasion; and (3) Sch B inhibits EMT of 4T1 cells as well as primary human breast cancer cells. We conclude that Sch B can attenuate 4T1 cell metastasis via inhibiting EMT.

Another possible mechanism for Sch B to inhibit metastasis could be its cytotoxic effect on 4T1 cells. This seems unlikely. Animal studies showed that Sch B did not significantly affect the growth of primary 4T1 tumors (Fig. 1, 2, 3), suggesting no cytotoxic effect. This is in general agreeable with the previous reports that this compound has low cytotoxicity toward cancer cells as compared with clinical anticancer drugs [17], [48], [49]. To further support that cytotoxicity did not play a major role in attenuating 4T1 cell metastasis, we included doxorubicin (Dox), a highly cytotoxic anticancer drug, in our experimental design. Although the cytotoxicity of Dox toward 4T1 is three orders of magnitude stronger than that of Sch B (data not shown), 4T1 lung metastases were significantly less in Sch B group than in the Dox or control group, but without significant difference between the latter two groups (Fig. S5A). Collectively, Sch B inhibiting 4T1 metastasis is unlikely via its cytotoxic effects.

While extending the life of cancer patients is the first priority in chemotherapy, the life quality of cancer patients is also very important. Many clinical anticancer drugs have severe side effects, such as neurotoxicity, gastrointestinal reaction, bone marrow inhibition, hair loss, etc., which substantially reduce the life quality of cancer patients. Hence, pain relief is increasingly recognized in cancer chemotherapy. On this regard, developing drugs that have very low systematic toxicity to human body would be more ideal. The safety of Sch B has been well documented. The previous reports from other laboratories indicated that in their protocols using Sch B to protect carbon tetrachloride-induced hepatotoxicity in mice, the dose of Sch B is 3 mmol/kg (1.2 g/kg), implicating the well-tolerance of this compound [50]. The toxicological data as reviewed by Hanke *et al.* revealed the high-safety of this compound [51]. In addition, there is a sufficient amount of evidence to show that this compound is safe to human. Sch B is the most abundant dibenzocyclooctadiene derivative in *S. chinensis* (Turcz.) Baill, a Chinese traditional medicinal herb. This herb has been used as a medication for the last several thousand years and is still being actively practiced in China, South Korea, and Japan, with no severe side effects ever reported.

Apart from its inhibition on cancer cell metastasis, Sch B combined with other anticancer drugs may bring other potential benefits. It has been reported that some anticancer agents might enhance metastasis via activating cancer cells’ invasiveness and metastasis. For examples, inhibition of angiogenesis reduced primary tumor growth and microvessel density, but accelerated tumor invasion and metastasis [52], [53]; cyclophosphamide and the Hsp90 inhibitor 17-AAG inhibited primary tumors but promoted tumor invasion and metastasis [54], [55]; doxorubicin promoted bone metastasis of 4T1 breast cancer cells [32]. We envision that combination of Sch B with these agents might bring benefit to control both primary tumor and metastasis.

## Supporting Information

Figure S1 **Effects of Sch B on lung metastasis of 4T1 cells.** Lung metastasis of 4T1 in mice demonstrated in Figure 2 were evaluated using H&E staining as described in Materials and Methods. The mice (n = 20 for each group) were sacrificed on day 30 and metastasis was quantified with H&E staining by counting the total tissue area per lung section and metastasis present in the same area. (A) The representative photos of visible surface lung nodules. (B) H & E staining of lung metastases. Under microscope, we were not able to take a complete picture with a single photo, so that we took several photos which were then combined.
(TIF)

Figure S2 **Effects of Sch B on MDA-MB-231 animal model.** 5×10^6^ viable MDA-MB-231 cells were inoculated s.c. into the second right mammary fat pad area. Mice were gavaged with Sch B (100 mg/kg body weight) every day for a total of 7 doses. Mice were sacrificed on day 30 after inoculation. Primary tumors, bones and surrounding tissues were pathologically examined using H&E. (A) The representative photos of invasive front, in which tumor cells invaded into the surrounding muscle in control group, while the invasion was attenuated in Sch B treated group. (B) Growth curves of primary tumor. (C) Body weight curves. (D) Tumor weight on the day of sacrifice. (E) Representative photos of lungs which have no visible metastatic nodules. (F) Leg bones (femora and tibiae) were fixed in 10% neutral buffered formalin, decalcified in 1% HCl for 24 hours and embedded in paraffin. Sections were stained with H&E, which shows no observable metastasis.
(TIF)

Figure S3 **The cytotoxicity of** **Sch B toward 4T1 cells.** 2×10^3^ cells were seeded per well in 96-well plates. Sch B (5, 10, 20, 40, 80, 160 µg/ml) was added and the cells were cultured for 60 hours. Cell viability was estimated by trypan blue exclusion assay. Six wells were measured for each concentration. Half inhibitory concentration (IC_50_) was determined.
(TIF)

Figure S4 **Sch B attenuates TGF-β induced migration and invasion of 4T1 cells** ***in vitro*** **.** 4T1 cells were treated with Sch B (5 µg/ml) and TGF-β (5 ng/ml) as described in Materials and Methods. (A) Representative photos of wound-healing assay that demonstrated TGF-β induced migration of 4T1 cells with or without pretreatment of Sch B (×40). (B) Representative photos of transwell assay showed TGF-β induced invasion of 4T1 cells with or without pretreatment of Sch B (×40).
(TIF)

Figure S5 **Effects of Sch B and doxorubicin (Dox) on 4T1 spontaneous metastasis mouse model.** 5×10^4^ viable 4T1 cells were inoculated *s.c.* in the second right mammary fat pad area to establish the spontaneously metastatic model. Mice were dosed with Sch B (100 mg/kg body weight) intragastrically, or injected with doxorubicin (2 mg/kg) i.p. every day for a total of 7 doses. Primary tumors were resected on day 10. Note that this is the same experiment illustrated in the Fig. 4, in which the data of Dox group are not included. The mice (n = 8 for each group) were sacrificed on day 30 and lung metastases were counted. (A) Lung metastases *, P<0.05, Sch B versus Dox group, **, P<0.01, Sch B versus control group. (B) Growth curves of body weights. *, P<0.05, Sch B, Dox versus control group, **, P<0.01, Sch B, Dox versus control group.
(TIF)
