# Expression of Concern: Schisandrin B Attenuates Cancer Invasion and Metastasis Via Inhibiting Epithelial-Mesenchymal Transition

**DOI:** 10.1371/journal.pone.0275601

**Published:** 2022-09-29

**Authors:** The PLOS One Editors

After this article [1] was published, concerns were raised about the mouse tumor sizes reported in Fig 1. Specifically:

- The chart in Fig 1A of the article [1] appears to report tumor sizes of up to 3000 mm^3^, with a standard error of approximately 100 mm^3^.
- The charts in Fig 1B and 1C appear to show tumour burdens greater than 10% of body weight.

In response to queries about these experiments, the corresponding author provided individual-level body weight, tumour length, tumour width, tumour volume and tumour weight data (see S1–S2 Files), and has made the following statements about the animal health monitoring protocols and endpoints used in the study:

- The study was approved by the IACUC of Zhejiang University in 2009.
- Body weight was used as a ‘surrogate marker’ and there was no weight loss in either control or Sch B groups, but the body weight of the Sch B group increased more than that for the control group.
- When taking into account the tumour weight, body weight decreased between 18 and 26 days for the control group and decreased between 18 and 22 days for the Sch B group.
- When a difference in body weight between the two groups was observed, the mice were checked twice daily.
- Soft bedding was used to reduce rubbing to prevent mechanical damage to the tumours.
- The animals were monitored every day before day 24 after inoculation, and twice daily on day 24 through day 30. The monitoring protocol included:
  ○ General appearance: including food and water consumption, dehydration, abnormal posture, change in behaviour.
  ○ Tumour conditions: including mouse body weight, tumour size, tumour ulceration/necrosis.
  ○ Organ functions: including hypothermia, bloodstain and mucopurulent discharge from any orifice, response to stimulation, movement, breathing, diarrhoea, vomiting.
- Signs of ulcers, anaemia or organ damage were not observed during the experiment.
- All mice were euthanised at day 30 using CO_2_ as the experiment was terminated when there were mice with a tumour diameter equal to or exceeding 2 cm. The corresponding author stated that if the number of days was lower, the difference between the two groups would likely not have had statistical significance.
- The following humane endpoint criteria were set:
  ○ Tumour mass/body weight exceeds 15% or tumour length exceeds 2 cm. Tumour mass was estimated according to the formula (length x width^2^/2).
  ○ Rapid body weight loss of no more than 20%.
  ○ Tumours that exhibit necrosis/ulceration.
  ○ Performance status (with any of these conditions: inability to eat or drink, severe lethargy, severe balance or gait disturbance).
  ○ Signs of severe organ or system involvement (with any of these conditions: persistent hypothermia, bloodstain and mucopurulent discharge from any orifice, laboured breathing, severe diarrhea or vomiting).
- In order to observe metastatic nodules in target organs it was estimated that the period of time for tumour growth should be at least 29 days, and the inoculated tumours would likely exceed 1.5 cm in diameter or exceed 10% of body weight.
- The estimated tumour mass/body weight did not exceed 15% (but did exceed 10%).
- On day 30, three out of 40 mice had tumours more than 2 cm in length and so the experiment ended, and all mice were euthanised.
- Actual tumour mass/body weight exceeded 15% in 23 out of 40 mice. The corresponding author stated that these tumours grew internally and this dimension could not be measured by callipers during the experiment, and that they did not have access to imaging equipment at the time of the original experiments.
- The study carried on through 90 days for one third of the animals to collect survival data. For these animals the primary tumour was removed by surgical resection on day 10 after 4T1 inoculation. No tumour recurrence was observed at the tumour inoculation sites in subsequent observations. As the primary tumour was resected and body weight increased during the whole-time course, humane endpoint for the survival experiment refers to the performance status and signs of severe organ or system involvement as indicated above. All control group mice and nine out of ten Sch B group mice were euthanised when the humane endpoint criteria were met. The remaining Sch B group mouse was euthanised at day 90. The individual level body weight data for the survival experiments are provided in S2 File; the number marked in red is the body weight of the animals that met the humane endpoint criteria and were euthanised.

The corresponding author stated that the original ethics approval letter for the study is no longer available, but a copy of a letter from Zhejiang University outlining the animal experiment ethics application, approval number and subject name was provided.

The *PLOS ONE* Editors consulted with an expert in animal research methodology who assessed the article, the underlying data and the authors’ comments, and confirmed that the control group tumor sizes reported in this article appear to exceed community standards for humane endpoint limits in mouse tumor studies. They also noted that the animals’ weight range listed in the article indicates the mice are not adults and that the normal weight gain as the animals aged during the study, plus the tumor burden due to metastasis, would mask weight loss indicative of cachexia and poor general health which puts the use of body weight as a humane endpoint indicator into question. The consulting expert also stated that the animal observations do not appear to be focused on key changes indicative of animal pain and distress.

In light of the above concerns, the *PLOS ONE* Editors issue this Expression of Concern. The editors regret that these concerns were not addressed at the time of the original review process.

## Supporting information

S1 File Underlying individual-level data supporting the charts in Fig 1A–1C. (XLSX)

S2 File Underlying individual-level data supporting the charts in Figs 2C–2E and 3A–3C. (XLSX)
