# Supplementary figures and images for: Schisandrin B Attenuates Cancer Invasion and Metastasis Via Inhibiting Epithelial-Mesenchymal Transition
Source: PLoS One. 2012 Jul 25;7(7):e40480. doi: 10.1371/journal.pone.0040480 (PMC3405072; doi:10.1371/journal.pone.0040480)

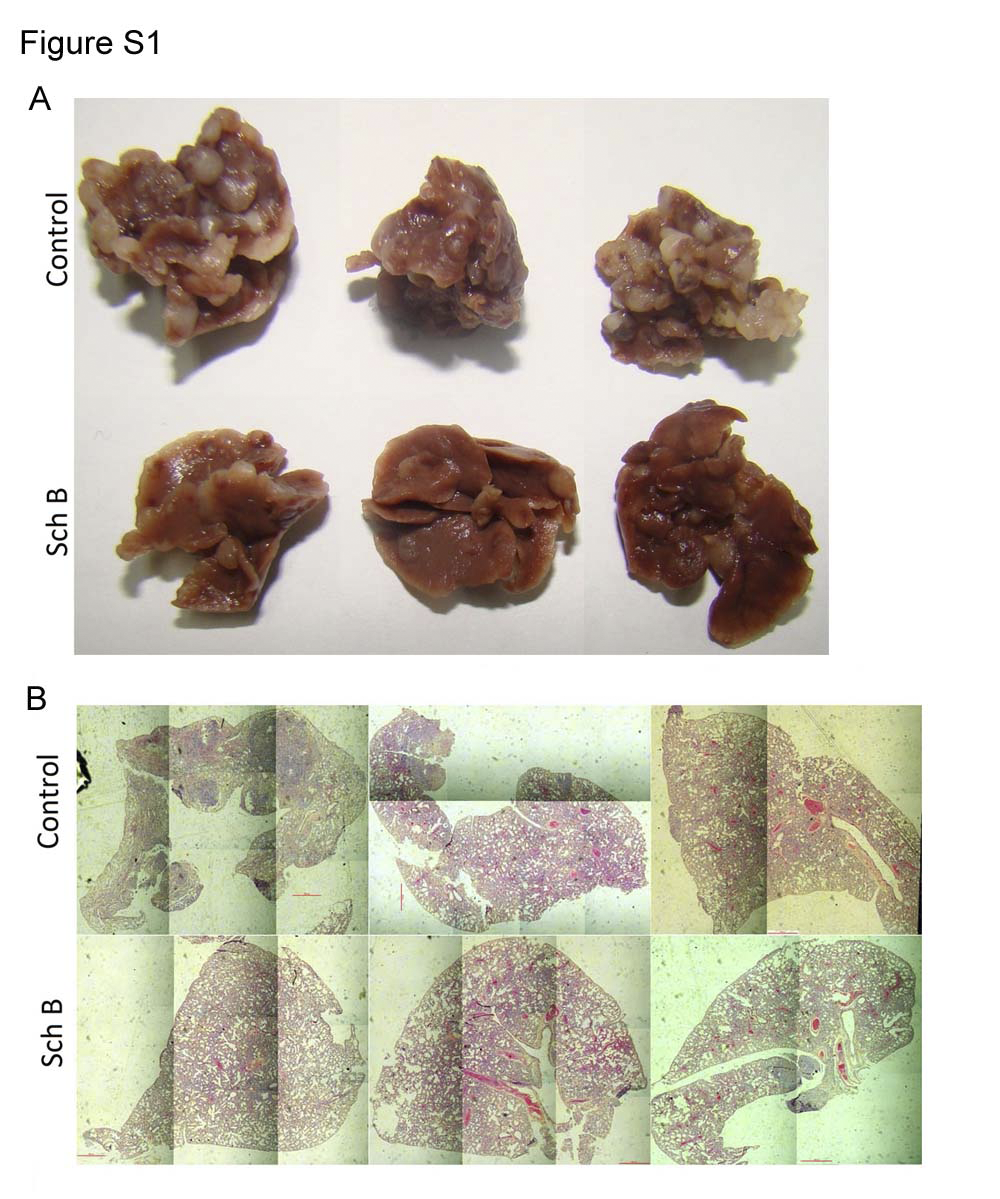

Supplement: Figure S1 — Effects of Sch B on lung metastasis of 4T1 cells. Lung metastasis of 4T1 in mice demonstrated in Figure 2 were evaluated using H&E staining as described in Materials and Methods. The mice (n = 20 for each group) were sacrificed on day 30 and metastasis was quantified with H&E staining by counting the total tissue area per lung section and metastasis present in the same area. (A) The representative photos of visible surface lung nodules. (B) H & E staining of lung metastases. Under microscope, we were not able to take a complete picture with a single photo, so that we took several photos which were then combined. (TIF) [file pone.0040480.s001.tif]

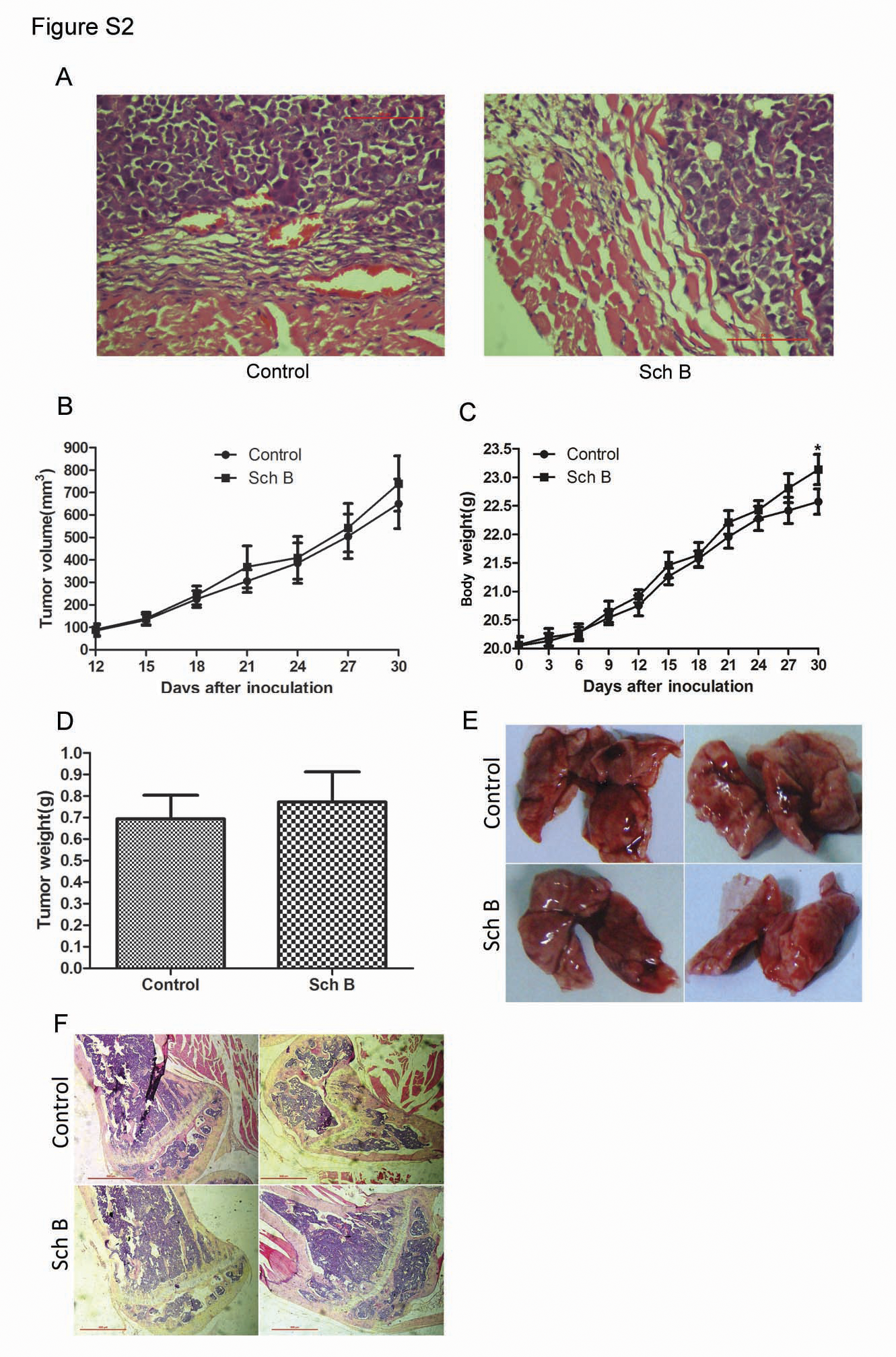

Supplement: Figure S2 — Effects of Sch B on MDA-MB-231 animal model. 5×10^6^ viable MDA-MB-231 cells were inoculated s.c. into the second right mammary fat pad area. Mice were gavaged with Sch B (100 mg/kg body weight) every day for a total of 7 doses. Mice were sacrificed on day 30 after inoculation. Primary tumors, bones and surrounding tissues were pathologically examined using H&E. (A) The representative photos of invasive front, in which tumor cells invaded into the surrounding muscle in control group, while the invasion was attenuated in Sch B treated group. (B) Growth curves of primary tumor. (C) Body weight curves. (D) Tumor weight on the day of sacrifice. (E) Representative photos of lungs which have no visible metastatic nodules. (F) Leg bones (femora and tibiae) were fixed in 10% neutral buffered formalin, decalcified in 1% HCl for 24 hours and embedded in paraffin. Sections were stained with H&E, which shows no observable metastasis. (TIF) [file pone.0040480.s002.tif]

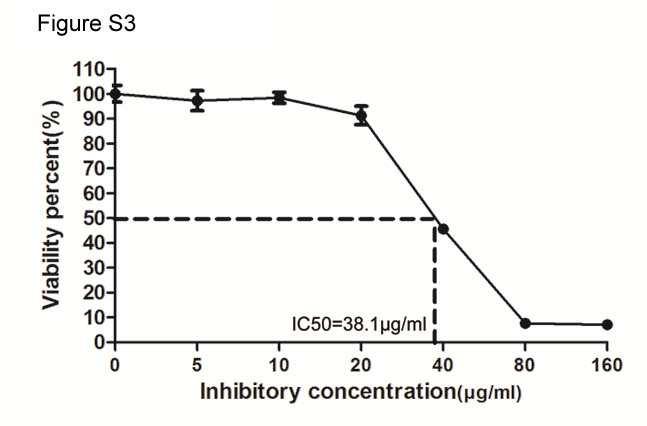

Supplement: Figure S3 — The cytotoxicity of Sch B toward 4T1 cells. 2×10^3^ cells were seeded per well in 96-well plates. Sch B (5, 10, 20, 40, 80, 160 µg/ml) was added and the cells were cultured for 60 hours. Cell viability was estimated by trypan blue exclusion assay. Six wells were measured for each concentration. Half inhibitory concentration (IC50) was determined. (TIF) [file pone.0040480.s003.tif]

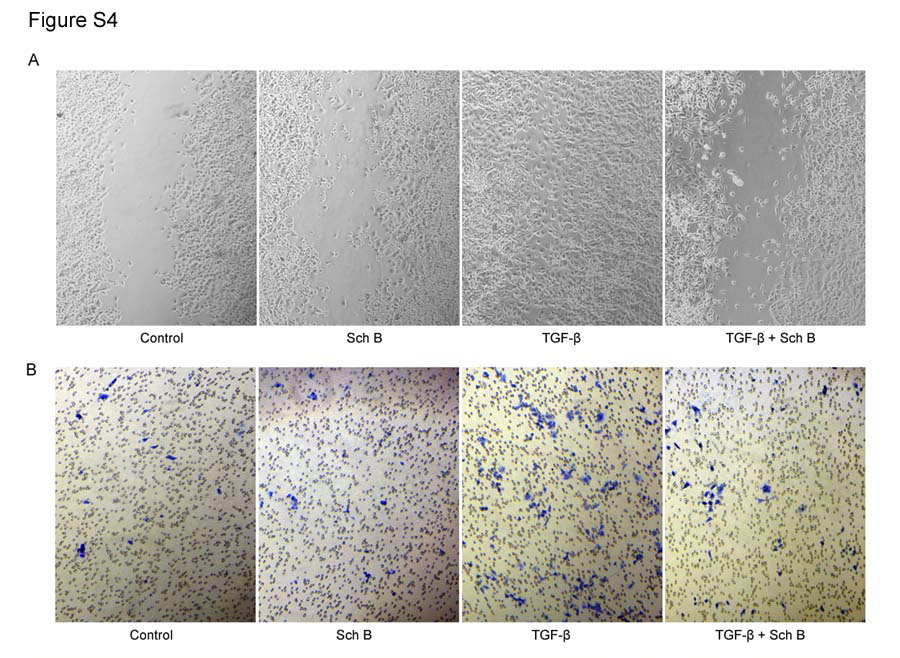

Supplement: Figure S4 — Sch B attenuates TGF-β induced migration and invasion of 4T1 cells in vitro . 4T1 cells were treated with Sch B (5 µg/ml) and TGF-β (5 ng/ml) as described in Materials and Methods. (A) Representative photos of wound-healing assay that demonstrated TGF-β induced migration of 4T1 cells with or without pretreatment of Sch B (×40). (B) Representative photos of transwell assay showed TGF-β induced invasion of 4T1 cells with or without pretreatment of Sch B (×40). (TIF) [file pone.0040480.s004.tif]

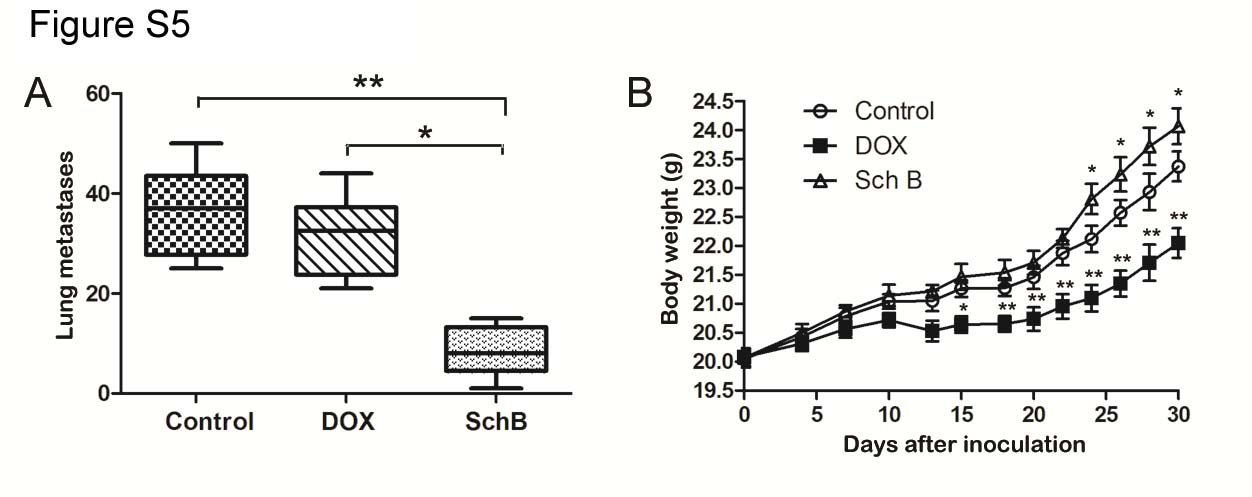

Supplement: Figure S5 — Effects of Sch B and doxorubicin (Dox) on 4T1 spontaneous metastasis mouse model. 5×10^4^ viable 4T1 cells were inoculated s.c. in the second right mammary fat pad area to establish the spontaneously metastatic model. Mice were dosed with Sch B (100 mg/kg body weight) intragastrically, or injected with doxorubicin (2 mg/kg) i.p. every day for a total of 7 doses. Primary tumors were resected on day 10. Note that this is the same experiment illustrated in the Fig. 4, in which the data of Dox group are not included. The mice (n = 8 for each group) were sacrificed on day 30 and lung metastases were counted. (A) Lung metastases *, P<0.05, Sch B versus Dox group, **, P<0.01, Sch B versus control group. (B) Growth curves of body weights. *, P<0.05, Sch B, Dox versus control group, **, P<0.01, Sch B, Dox versus control group. (TIF) [file pone.0040480.s005.tif]
